# Hypoventilation training including maximal end-expiratory breath holding improves the ability to repeat high-intensity efforts in elite judo athletes

**DOI:** 10.3389/fphys.2024.1441696

**Published:** 2024-09-27

**Authors:** X. Woorons, C. Faucher, S. P. Dufour, F. Brocherie, P. Robach, P. Connes, J. V. Brugniaux, S. Verges, A. F. Gaston, G. Millet, O. Dupuy, A. Pichon

**Affiliations:** ^1^ Univ. Lille, Univ. Artois, Univ. Littoral Côte d’Opale, ULR 7369 - URePSSS - Unité de Recherche Pluridisciplinaire Sport Santé Société, Lille, France; ^2^ Association for Research and Promotion of Hypoventilation Training (ARPEH), Lille, France; ^3^ Laboratoire MOVE UR 20296 - UR, Faculté des Sciences du Sport-STAPS, Université de Poitiers, Poitiers, France; ^4^ Centre d’Investigation Clinique CIC 1402, Université de Poitiers, CHU Poitiers, INSERM, Poitiers, France; ^5^ Faculty of Sport Sciences, CEERIPE (UR3072), University of Strasbourg, Strasbourg, France; ^6^ Laboratory Sport, Expertise, and Performance (EA 7370), French Institute of Sport (INSEP), Paris, France; ^7^ Ecole Nationale des Sports de Montagne, site de l’Ecole Nationale de Ski et d’Alpinisme, Chamonix, France; ^8^ Laboratory LIBM (UR7424), Vascular Biology and Red Blood Cell team, University of Lyon, Lyon, France; ^9^ HP2 laboratory, Univ. Grenoble Alpes, INSERM, CHU Grenoble Alpes, Grenoble, France; ^10^ Laboratory LIPSEM (UR 4604), University of Perpignan Via Domitia, Font-Romeu, France; ^11^ Institute of Sport Sciences, University of Lausanne, Lausanne, Switzerland; ^12^ École de Kinésiologie et des Sciences de l’activité physique (EKSAP), Faculté de Médecine, Université de Montréal, Montréal, QC, Canada

**Keywords:** hypoxia, hypercapnia, breath holding, repeated-sprint ability, training

## Abstract

**Purpose:**

To investigate the effects of a repeated-sprint training in hypoxia induced by voluntary hypoventilation at low lung volume (RSH-VHL) including end-expiratory breath holding (EEBH) of maximal duration.

**Methods:**

Over a 4-week period, twenty elite judo athletes (10 women and 10 men) were randomly split into two groups to perform 8 sessions of rowing repeated-sprint exercise either with RSH-VHL (each sprint with maximal EEBH) or with unrestricted breathing (RSN, 10-s sprints). Before (Pre-), 5 days after (Post-1) and 12 days after (Post-2) the last training session, participants completed a repeated-sprint ability (RSA) test on a rowing ergometer (8 × 25-s “all-out” repetitions interspersed with 25 s of passive recovery). Power output (PO), oxygen uptake, perceptual-motor capacity (turning off a traffic light with a predetermined code), cerebral (Δ[Hb_diff_]) and muscle (Δ[Hb/Mb]_diff_) oxygenation, cerebral total haemoglobin concentration (Δ[THb]) and muscle total haemoglobin/myoglobin concentration (Δ[THb/Mb]) were measured during each RSA repetition and/or recovery period.

**Results:**

From Pre-to Post-1 and Post-2, maximal PO, mean PO (MPO) of the first half of the test (repetitions 1–4), oxygen uptake, end-repetition cerebral Δ[Hb_diff_] and Δ[THb], end-repetition muscle Δ[Hb/Mb]_diff_ and Δ[THb/Mb] and perceptual-motor capacity remained unchanged in both groups. Conversely, MPO of the second half of the test (repetitions 5–8) was higher at Post-1 than at Pre-in RSH-VHL only (*p* < 0.01), resulting in a lower percentage decrement score over the entire RSA test (20.4% ± 6.5% vs. 23.9% ± 7.0%, *p* = 0.01). Furthermore, MPO (5–8) was greater in RSH-VHL than in RSN at Post-1 (*p* = 0.04). These performance results were accompanied by an increase in muscle Δ[THb/Mb] (p < 0.01) and a concomitant decrease in cerebral Δ[THb] (*p* < 0.01) during the recovery periods of the RSA test at Post-1 in RSH-VHL.

**Conclusion:**

Four weeks of RSH-VHL including maximal EEBH improved the ability of elite judo athletes to repeat high-intensity efforts. The performance improvement, observed 5 days but not 12 days after training, may be due to enhanced muscle perfusion. The unchanged oxygen uptake and the decrease in cerebral regional blood volume observed at the same time suggest that a blood volume redistribution occurred after the RSH-VHL intervention to meet the increase in muscle perfusion.

## 1 Introduction

Among the different altitude/hypoxic training methods, the “Living Low-Training High” approach (LLTH) has gained in popularity over the last decade. One of the advantages of LLTH is that it limits the constraints for athletes in terms of logistics and time of exposure to hypoxia (generally < 2 h). The LLTH approach can be implemented through passive (i.e., ischemic pre-conditioning and intermittent hypoxic exposure at rest) or active (e.g., interval hypoxic training, repeated-sprint training in hypoxia) modalities (Girard et al., 2020).

Recently, a new active modality, the so-called voluntary hypoventilation at low lung volume (VHL), has been included in the nomenclature of the LLTH methods (Girard et al., 2017). The VHL technique, which consists of repeating short bouts of exercise (generally 4–6 s) with end-expiratory breath holding (EEBH), has been consistently reported to induce a drop in peripheral arterial oxygen saturation (SpO_2_) (i.e., down to 86%–88% on average) and in muscle oxygenation as well as higher levels of blood and pulmonary carbon dioxide partial pressures as compared with the same exercise performed with unrestricted breathing (Kume et al., 2016; Toubekis et al., 2017; Woorons et al., 2007; Woorons et al., 2008; Woorons et al., 2010; Woorons et al., 2014; Woorons et al., 2017). After several weeks of high-intensity VHL training, significant gains in anaerobic performance (Trincat et al., 2017; Woorons et al., 2016; Woorons et al., 2019a) and in repeated-sprint ability (RSA) (Ait Ali Braham et al., 2024; Brocherie et al., 2022; Fornasier Santos et al., 2018; Lapointe et al., 2020; Trincat et al., 2017; Woorons et al., 2019a; Woorons et al., 2020) have been reported in athletes of different sporting disciplines.

Since 2021, a more ambitious approach of the VHL method, combining high or supramaximal exercise intensities with maximal EEBH has been acutely investigated in several studies (Woorons et al., 2021a; Woorons et al., 2021b; Woorons et al., 2023). Under this condition, dramatic drops of SpO_2_ have been recorded (i.e., down to 73%–75%) and were accompanied by large and early decline in muscle oxygenation (Woorons et al., 2021b; Woorons et al., 2023). A marked increase in left ventricular stroke volume (Woorons et al., 2021a) as well as in muscle and cerebral blood volume (Woorons et al., 2023) were also reported. However, the impact of this specific maximal EEBH approach on performance and the physiological adaptations it may induce after a period of training remain to be investigated.

So far, it has been shown that performing a repeated-sprint training in hypoxia induced by VHL (RSH-VHL) with EEBH of 6-s duration could lead to higher oxygen uptake (Woorons et al., 2019a) and greater muscle reoxygenation (Lapointe et al., 2020) during a RSA test in cyclists and basketball players, respectively after 3 and 4 weeks of training. However, in these studies, no improvement in muscle oxygen utilization or in muscle perfusion was found. These results may be different when the hypoxic stimulus is enhanced through maximal EEBH. Furthermore, the influence of VHL training on cerebral oxygenation, which has been shown to play a role in motor fatigue and cognitive performance during strenuous exercise (Mekari et al., 2015), has yet to be investigated. Another point is that only a single post-test (ranging from 2 days to 1 week after the end of the training period) was implemented in the previous studies investigating the chronic effects of VHL (Brocherie et al., 2022; Fornasier-Santos et al., 2018; Trincat et al., 2017; Woorons et al., 2019a). It remains unknown whether a change in performance and/or in physiological parameters could still occur, or even be greater, more than 1 week after the end of the training period. Finally, while high-intensity VHL training has been shown to be effective in highly-trained athletes up to national level, its efficacy in elite athletes, who generally have less room for performance improvement, remains to be demonstrated.

Therefore, the main objective of the present study was to investigate the effects of 4 weeks of RSH-VHL including maximal EEBH on RSA performance and on muscle and cerebral oxygenation in elite judo athletes. We aimed to assess these effects within the first and the second week following the end of the training period. We hypothesized that the RSH-VHL intervention would improve RSA to a greater extent than similar training with unrestricted breathing. We also assumed that the increase in performance after RSH-VHL would be accompanied by greater muscle oxygen utilization, enhanced muscle perfusion and possibly improved cerebral oxygenation.

## 2 Methods

### 2.1 Participants

Twenty elite judo athletes (10 women and 10 men) belonging to a French National Center were selected to participate in this study. Participants were engaged in competition at national and international levels and reported at least 10 years of judo practice at the time of the experiment. Their weekly training volume was about 20 h (11–13 sessions per week). Participants belonged to two different training groups, male and female, which followed the same training program within each group during the study. The main characteristics of the participants are presented in Table 1. All of the participants were sea level residents (i.e., < 500 m) and were not acclimatized or recently exposed to altitude or hypoxia before and during the experiment. Furthermore, none of them had ever used any hypoxic or VHL training methods before the study. The participants gave their written informed consent to participate in this study which was approved by the French Ethics Committee for Research in Sports Science (2019-22-02-32) and complied with the Declaration of Helsinki (2008).

**TABLE 1:** Characteristics of the subjects.

	n	Age (y)	Height (cm)	Weight (kg)
RSH-VHL				
Women	5	21.2 ± 3.3	160.4 ± 8.1	55.0 ± 9.6
Men	5	20.0 ± 1.4	174.4 ± 8.4	69.5 ± 8.4
Women + men	10	20.6 ± 2.5	167.4 ± 11	62.3 ± 11
RSN				
Women	5	19.2 ± 1.3	165.8 ± 7.7	59.6 ± 5.5
Men	5	20.0 ± 0.7	171.4 ± 3.7	69.4 ± 9.3
Women + men	10	19.6 ± 1.1	168.6 ± 6.4	64.5 ± 8.8

RSH-VHL, repeated-sprint training in hypoxia induced by voluntary hypoventilation at low lung volume; RSN, repeated-sprint training with unrestricted breathing. Values for age, height and weight are expressed as mean ± SD.

### 2.2 Study design

**FIGURE 1:**
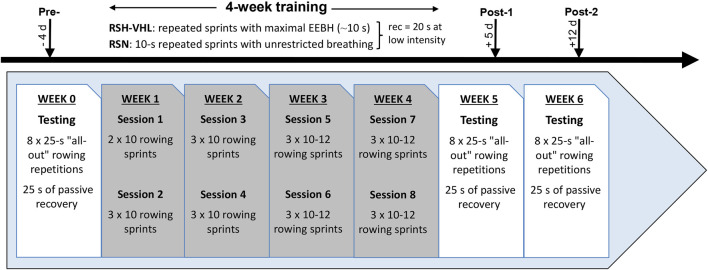
Experimental design of the study. RSH-VHL, repeated-sprint training with voluntary hypoventilation at low lung volume; EEBH, end-expiratory breath holding; RSN, repeated-sprint training with unrestricted breathing; rec, recovery; d, days.

The experimental protocol (Figure 1) consisted of performing 8 supervised training sessions of repeated “all-out” rowing exercises (i.e., repeated sprints) over a 4-week period. Four days before (Pre-), 5 days after (Post-1) and 12 days after (Post-2) the training period, a testing session was carried out on a rowing ergometer for the assessment of RSA and the measurement of physiological parameters. The choice to use a rowing ergometer for this study was made in agreement with the coaches of the French Judo Federation based on the fact that the movements are specific to judo. Over the 4 weeks prior to the start of the experiment, participants were asked to perform two weekly 30-min sessions of rowing exercises (one at moderate intensity and the other at high intensity). They were therefore familiarized to this type of exercise when they carried out the first testing session. All training and testing sessions were performed at sea level (normoxia), in an air-conditioned room with an ambient temperature maintained at 18C°–19°C. Participants were matched into pairs for gender and their performance level (i.e., mean power output) recorded in the rowing high-intensity sessions preceding the experiment. They were then randomly assigned by drawing lots within each pair to a group that performed the rowing repeated-sprint exercises either in hypoxia induced by VHL (RSH-VHL, n = 10) or with unrestricted breathing (RSN, n = 10). Before the start of the experiment, participants were familiarized with the testing procedures and with the VHL technique which has been used and described previously (Woorons et al., 2017).

### 2.3 Testing

The RSA test consisted of eight “all-out” 25-s repetitions (with unrestricted breathing) interspersed by 25 s of passive recovery performed on a rowing ergometer using an air-braked resistance mechanism generated by a spinning flywheel (Concept 2, Inc., Morrisville, United States). The air resistance of the rowing ergometer, which ranges from 1 to 10, was set relative to each participant’s body weight. Before testing, a standardized warm-up (i.e., 10 min of exercise at low-to-moderate intensity and two 10-s sprints at maximal intensity) was implemented. After a 10-min resting period, during which the experimental equipment was placed on the participants, a single “all-out” 25-s repetition was performed to determine the reference mean power output (MPO_ref_). It was followed by 5 min of rest before starting the RSA test. In the first repetition of the test, participants were required to achieve at least 90% of MPO_ref_. If they failed to do so, they had to restart the RSA test after a 5-min period of rest. All participants were strongly encouraged to maintain the highest intensity during each repetition. Within the 24 h preceding the tests, they were instructed to avoid high-intensity training and to refrain from consuming caffeine and alcohol. Each participant performed the same test at the same time and day before and after the training period.

### 2.4 Training

Over a 4-week period, participants had to complete eight repeated-sprint sessions (two per week separated by 48–72 h) on the same rowing ergometer and with the same resistance as for the testing sessions. Training included the same 10-min warm-up as for the testing sessions. In the RSH-VHL group, participants were required to perform each sprint with maximal EEBH. This specific VHL approach has been used and described in previous studies (Woorons et al., 2021a; Woorons et al., 2021b; Woorons et al., 2023). Briefly, at the start of each sprint, participants had to do a first exhalation down to around the functional residual capacity (i.e., “normal exhalation”) then to row at maximal intensity while maintaining EEBH for as long as possible. Each sprint ended at the release of EEBH (i.e., at the breaking point) with a second exhalation down to the residual volume to empty the air remaining in the lungs. It was then followed by 20 s of semi-active recovery (rowing at very low intensity) with unrestricted breathing. In the preparation phase of this study, we observed that EEBHs could be maintained for ∼10 s. Therefore, in the RSN group, participants were required to perform 10-s rowing sprints at maximal intensity and with unrestricted breathing. In this group, the start of the sprints was given every 30 s (same duration and modality of recovery as in the RSH-VHL group). In the first session, both groups had to complete two sets of 10 sprint repetitions. From the 2^nd^ to the 4^th^ session, they completed 3 sets of 10 sprints. Over the next four sessions, the number of sprints per set was progressively increased according to the participants’ rate of perceived exertion (RPE, 10-point Borg scale), reaching a maximum of 12 repetitions per set in the last session. Each set was separated by 3 min of active recovery (i.e., rowing at low intensity).

### 2.5 Measurements

#### 2.5.1 Training

All training sessions were supervised by the same experimenter. For each participant, the number of sets and sprints per set, as well as the end-exercise RPE were measured at each session. During two repeated-sprint sessions including three sets, power output was measured (PM3 Performance Monitor, Concept 2, Inc., Morrisville, United States) and averaged over the duration of each sprint. Furthermore, SpO_2_ was continuously recorded (Nellcor PM10-N, Pleasanton, CA, United States) in all participants of both groups. In addition, for the RSH-VHL group, the duration of each sprint with EEBH was measured.

#### 2.5.2 Testing

##### 2.5.2.1 Performance

Power output was measured with the PM3 Performance Monitor of the Concept 2 ergometer (Inc., Morrisville, United States) and averaged over each 25-s repetition (MPO, expressed in watts) for the analyses. The maximal MPO (MPO_max_) reached in the RSA test was determined and the average MPO over the 8 repetitions (MPO_mean(1–8)_) was calculated in absolute (i.e., watts) and in relative values (i.e., percentage of MPO_ref_) as previously done (Fornasier-Santos et al., 2018; Brocherie et al., 2022). Given that most of the previous RSH-VHL studies using a closed-loop RSA test reported a greater performance improvement in the second than in the first part of the test (Ait Ali Braham et al., 2024; Brocherie et al., 2022; Lapointe et al., 2020; Woorons et al., 2019a), we also analysed separately the mean MPO over repetitions 1–4 (MPO_mean(1–4)_) and over repetitions 5–8 (MPO_mean(5–8)_). RPE was assessed at the end of the RSA test. Fatigue was also evaluated through the percentage decrement score (S_dec_) as follows.

$$│\left(100\;*\;\left(\text{total repetitions MPO}/\text{ideal repetitions MPO}\right)\right)\;-\;100│$$

Where total repetitions MPO is the sum of the 8 repetitions MPO (in watts) and ideal repetitions MPO is the number of repetitions (i.e., 8) × highest MPO (in watts) reached in the single 25-s repetition or in the RSA test.

This formula has been found to be the most valid and reliable method for quantifying fatigue in tests of multiple-sprint performance (Glaister et al., 2008).

##### 2.5.2.2 Perceptual-motor capacity

Perceptual-motor capacity, which is an important component during a judo match, was evaluated during the recovery periods of the RSA test after the second, fourth, sixth and eight repetition (WittySem device, Microgate, United States). Four traffic lights were used and positioned in pairs (one high and one low) on either side of the rowing ergometer. Each test started immediately after the end of the repetitions and lasted 20 s. It consisted of catching (i.e., turning off) as many times as possible the traffic light randomly displaying a predetermined code by approaching the proximity sensor with a hand. The total number of hits was measured for each test.

##### 2.5.2.3 Gas exchange and heart rate

After performing the standardized calibration procedures, gas exchange was continuously recorded during the entire RSA test with a breath-by-breath portable system (K4b^2^, Cosmed, Rome, Italy). Oxygen uptake ( $\dot{V}O_{2}$ ), ventilation ( $\dot{V}E$ ), end-tidal partial pressures in oxygen (PETO_2_) and in carbon dioxide (PETCO_2_) were measured. Heart rate (HR) was also measured with the same device via an association with a Polar T34 strap (Polar Electro Inc., Lake Success, NY, United States) placed on the chest of participants. We also calculated oxygen pulse ( $\dot{V}O_{2}$ /HR) which provides an index of stroke volume during steady-state submaximal exercise (Whipp et al., 1996) but might also be used during repeated-sprint exercise (Woorons et al., 2019a). As reported elsewhere (Lapointe et al., 2020; Woorons et al., 2019a), we chose to analyse data over both the 25-s repetitions and the 25-s recovery periods of the RSA test.

##### 2.5.2.4 Arterial oxygen saturation

SpO_2_ was measured throughout the RSA test with the pulse oximeter Nellcor PM10-N (Pleasanton, CA, United States) and an adhesive forehead sensor (Max-Fast, Nellcor, Pleasanton, United States) which was positioned above the left orbital area. An adjustable headband was placed over the forehead sensor to ensure gentle, consistent pressure on the sensor device. SpO_2_ was recorded in real time second per second and collected using a data acquisition system (Score Analysis Software, Nellcor, Pleasanton, United States). Data were averaged over 2-s periods and the values were analyzed in the last 2 s of each repetition and recovery period.

##### 2.5.2.5 Near-infrared spectroscopy

Cerebral and muscle oxygenation were assessed using the near-infrared spectroscopy (NIRS) technique (Boushel and Piantadosi, 2000) at wavelengths between 760 and 850 nm. For cerebral oxygenation, we used the PortaLite device (Artinis Medical Systems, Einsteinweg, Netherlands) which position was standardized on the surface of the right prefrontal cortex (above the eyebrow, between the midline of the skull and the *temporalis* muscle) (Billaut et al., 2013; Willis et al., 2017; Woorons et al., 2019b). The probe was secured with a double-sided tape and was placed under the headband used for maintaining the forehead sensor of the pulse oximeter. An age-dependent differential pathlength factor was used (Duncan et al., 1996). For muscle oxygenation, we used the PortaMon device (Artinis Medical Systems, Einsteinweg, Netherlands) which was placed at the lower third of the right-leg *vastus lateralis* muscle, parallel to the long axis of the muscle. A differential pathlength factor of 4.0 was used. The probe was attached to the skin with a double-sided tape and firmly fastened with an opaque cotton elastic band wrapped around participants’ thigh. Position of the probe was marked at the first testing session with a permanent pen. Participants were asked to maintain this mark during the whole protocol for accurate repositioning at the following testing sessions. For both cerebral and muscle NIRS measurements, we used the greatest distance between the receiver and transmitters (i.e., interoptode spacing of 40 mm). All NIRS signals were acquired with the *OxySoft* software (*Artinis* Medical Systems, Elst, Netherlands) and recorded with a sampling frequency of 10 Hz. A 10^th^-order low-pass zero-phase Butterworth filter (cut-off frequency 0.1 Hz) was applied to reduce artefacts and smooth the signal perturbations (Woorons et al., 2019a).

Concentrations of oxyhaemoglobin ([O_2_Hb]) and deoxyhaemoglobin ([HHb]) were recorded at the cerebral level while muscle oxy-haemoglobin/myoglobin ([O_2_Hb/Mb]) and deoxy-haemoglobin/myoglobin ([HHb/Mb]) concentrations were measured. The sum of [O_2_Hb] and [HHb] (i.e [THb]) and of [O_2_Hb/Mb] and [HHb/Mb] (i.e., [THb/Mb]) were calculated to obtain cerebral total haemoglobin and muscle total haemoglobin/myoglobin respectively which were used as indices of regional blood volume. To analyse cerebral and muscle oxygenation, we used the ‘‘oxygenation indices” [Hb]_diff_ (i.e., [O_2_Hb] - [HHb]) and [Hb/Mb]_diff_ (i.e [O_2_Hb/Mb] - [HHb/Mb]) respectively. These indices have been used for long (Grassi et al., 2003; MacDonald et al., 1999) and are more reliable and relevant than [O_2_Hb] or [HHb] when total haemoglobin is not constant (van Beekvelt et al., 2002). Positive and negative values of these indices represent the degree of oxygenation and deoxygenation, respectively. The change (Δ) in cerebral [THb] and [Hb]_diff_ and in muscle [THb/Mb] and [Hb/Mb]_diff_ were assessed from the resting values (seated position) recorded over the last minute preceding the start of the test. The measurements were therefore normalized from these recordings (arbitrarily defined as 0 µM). For all variables, data were averaged over 2 s and analysed in the last 2 s of each repetition and each recovery period. To assess muscle reoxygenation capacity, we calculated the difference in Δ[THb/Mb] (i.e., Reoxy [THb/Mb]) and in Δ[Hb/Mb]_diff_ (i.e., Reoxy [Hb/Mb]_diff_) between the end of each recovery period and the end of the previous repetition (last 2 s for both) (Billaut and Buchheit, 2013).

### 2.6 Statistics

All data are expressed as mean ± SD and were first tested for distribution normality and variance homogeneity. The effect of condition (i.e., training intervention, RSH-VHL vs. RSN) and time (Pre-vs. Post-1 vs. Post-2) on the mean of the variables measured in the testing sessions were analysed using mixed two-way ANOVA for repeated-measures. We also used mixed two-way ANOVA for repeated measures to analyse the change in MPO, $\dot{V}O_{2\;}$ and NIRS data throughout the RSA test (i.e., repetition by repetition) within each group (time x repetition number separately for RSH-VHL and RSN) and between groups (condition x repetition number for Pre-, Post-1 and Post-2 separately). When a significant main effect was found, irrespective of an interaction effect, the Bonferroni *post hoc* test was performed to localize the difference as recommended by Wei et al., 2012 and as applied in previous studies in the area of biology (Jobgen et al., 2009; Lassala et al., 2010; Tan et al., 2010) or dealing with the RSH-VHL method (Ait Ali Braham et al., 2024; Brocherie et al., 2022; Fornasier Santos et al., 2018). The comparisons between groups in the variables measured during training were made with Student T-tests. All analyses were performed with Sigmastat 4.0 software (Systat Software, CA, United States). Null hypothesis was rejected at *p* < 0.05.

## 3 Results

### 3.1 Training

Training data are presented in Table 2. One participant in the RSH-VHL group and two participants in the RSN group missed one training session. All the remaining participants completed the 8 training sessions. There was no difference between groups in the total number of sprints over the 4-week training period (*p* = 0.76). The measurements made over two training sessions show that the mean power output per sprint was also not different between groups (*p* = 0.78). On the other hand, RPE was higher in RSH-VHL than in RSN (*p* < 0.01) whereas the mean minimal SpO_2_ per sprint (*p* < 0.01) and the mean nadir SpO_2_ per set (*p* < 0.01) were lower in the former than in the latter condition. In RSH-VHL, the mean time per sprint, which corresponded to the average time of the maximal EEBH, was lower than in RSN (*p* = 0.02).

**TABLE 2:** Training data.

	RSH-VHL	RSN
Total number of training sessions (n)	7.9 ± 0.3	7.8 ± 0.4
Mean RPE per training session	8.7 ± 0.4*	7.9 ± 0.5
Total number of sprints (n)	233 ± 14	235 ± 15
Mean power output per sprint (W)	281 ± 102	293 ± 81
Mean time per sprint (s)	9.4 ± 0.7*	10.0 ± 0.2
Mean minimal SpO_2_ per sprint (%)	78.7 ± 7.1*	96.6 ± 0.5
Mean nadir SpO_2_ per set (%)	71.1 ± 9.3^*^	95.6 ± 0.9

RSH-VHL, repeated-sprint training in hypoxia induced by voluntary hypoventilation at low lung volume; RSN, repeated-sprint training with unrestricted breathing; RPE, rate of perceived exertion; SpO_2_, arterial oxygen saturation. * Significantly different from RSN., Values are expressed as mean ± SD.

### 3.2 Testing

#### 3.2.1 Performance

**FIGURE 2:**
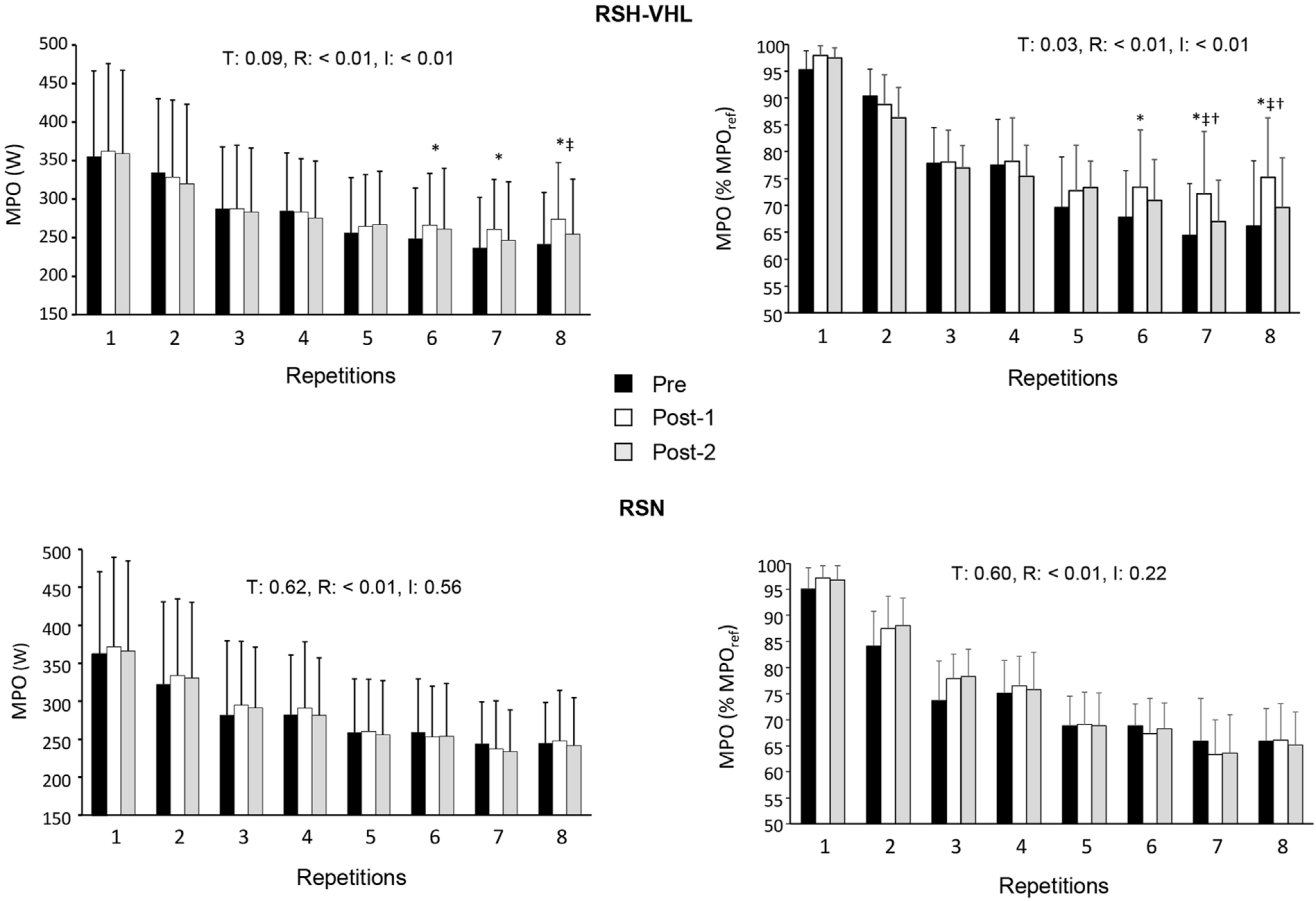
Mean power output (MPO) expressed in watts (W, left panels) and in percentage of the reference MPO (MPO_ref_, right panel) for each repetition of the repeated-sprint ability test before (Pre-), 5 days after (Post-1) and 12 days after (Post-2) the repeated-sprint training in hypoxia induced by voluntary hypoventilation at low lung volume (RSH-VHL, upper panels) and with unrestricted breathing (RSN, lower panels). T: ANOVA time effect (Pre-vs. Post-1 vs. Post-2); R: ANOVA repetition effect; I: ANOVA interaction effect. *, significant difference with the same sprint at Pre-; ^‡^, significant difference with the same sprint at Post-2; ^†^ significant difference with RSN for the same sprint at Post-1; *p* < 0.05.

Data are presented in Table 3 and Figure 2.

**Table 3:** Results of performance and perceptual-motor capacity in the RSA test before (Pre-), 5 days after (Post-1), and 12 days after (Post-2) the training period.

	**RSH-VHL**			**RSN**			**ANOVA *P* value**		
	Pre-	Post-1	Post-2	Pre-	Post-1	Post-2	T	C	T × C
**Performance**									
25-s MPO_ref_ (W)	370 ± 106	370 ± 114	368 ± 109	378 ± 103	381 ± 114	377 ± 115	0.80	0.85	0.93
MPO_max_ (W)	357 ± 109	362 ± 113	359 ± 108	362 ± 109	373 ± 114	367 ± 116	0.35	0.87	0.89
MPO_mean(1-8)_ (W)	281 ± 76	291 ± 77	283 ± 82	282 ± 79	286 ± 80	282 ± 77	0.09	0.96	0.72
MPO_mean(1-4)_ (W)	315 ± 89	315.3 ± 90	309 ± 91	312 ± 97	323 ± 96	318 ± 92	0.32	0.92	0.30
MPO_mean(5-8)_ (W)	246 ± 66	**266 ± 67** ^*^	257 ± 73	252 ± 61	250 ± 66	246 ± 64	0.07	0.81	**0.02**
MPO_mean(1-8)_ (% MPO_ref_)	76.1 ± 7.0	**79.6 ± 6.5** ^*^	77.1 ± 4.8	74.7 ± 3.0	75.6 ± 4.2	75.6 ± 4.4	**0.03**	0.29	0.21
MPO_mean(1-4)_ (% MPO_ref_)	85.2 ± 4.7	85.7 ± 3.8	84.0 ± 3.3	82.0 ± 4.2	84.7 ± 3.1	84.8 ± 3.6	0.17	0.41	0.08
MPO_mean(5-8)_ (% MPO_ref_)	67.0 ± 10	**73.4 ± 10** ^*†^	70.2 ± 6.9	67.4 ± 5.4	66.5 ± 6.4	66.4 ± 5.9	0.06	0.28	**< 0.01**
S_dec_ (%)	23.9 ± 7.0	**20.4 ± 6.5** ^*^	22.8 ± 4.8	25.1 ± 2.9	24.4 ± 4.2	24.4 ± 4.4	**0.04**	0.30	0.18
RPE	9.1 ± 0.4	**8.5 ± 0.4** ^*‡†^	8.9 ± 0.4	8.7 ± 0.5	9.0 ± 0.6	9.0 ± 0.5	**< 0.01**	0.47	**< 0.01**
**Perceptual-motor capacity** (mean number of hits)									
Test 1 (recovery 2)	15.8 ± 2.2	14.5 ± 1.4	14.5 ± 2.8	15.3 ± 1.7	13.8 ± 1.7	14.1 ± 2.0	**< 0.01**	0.47	0.95
Test 2 (recovery 4)	14.6 ± 2.2	14.7 ± 2.1	14.9 ± 2.5	14.7 ± 1.1	14.4 ± 1.7	13.9 ± 1.4	0.78	0.59	0.31
Test 3 (recovery 6)	14.0 ± 2.3	14.5 ± 1.9	15.0 ± 1.7	13.7 ± 1.3	14.2 ± 1.6	14.4 ± 1.5	**0.03**	0.57	0.86
Test 4 (recovery 8)	13.4 ± 2.2	**15.3 ± 2.1** ^*^	**15.5 ± 1.8** ^*^	12.6 ± 1.0	**15.1 ± 1.6** ^*^	**14.2 ± 1.5** ^*^	**< 0.01**	0.27	0.24
Mean of 4 tests	14.5 ± 2.1	14.8 ± 1.7	15.0 ± 2.0	14.1 ± 1.1	14.4 ± 1.4	14.2 ± 1.3	0.37	0.44	0.57

Values are expressed as mean ± SD.

RSA, repeated-sprint ability; RSH-VHL, repeated-sprint training in hypoxia induced by voluntary hypoventilation at low lung volume; RSN, repeated-sprint training with unrestricted breathing; MPO_ref_, reference mean power output obtained in the single 25-s repetition; MPO_max_, maximal mean power output of the RSA test; MPO_mean(1-8)_, average mean power output of the repetitions 1 to 8 of the RSA test; MPO_mean(1-4)_, average mean power output of the repetitions 1 to 4 of the RSA test; MPO_mean(5-8)_, average mean power output of the repetitions 5 to 8 of the RSA test; Sdec, percentage decrement score; RPE, rate of perceived exertion; T, time effect; C, condition effect; T x C, interaction effect (time x condition); bold values show significant difference. *, significantly different from Pre- within group; ^‡^ significantly different from Post-2 within group; † significantly different from RSN for the same testing session; *p* < 0.05.

There was no time (Pre-vs. Post-1 vs. Post-2) or condition effect for MPO_ref_, MPO_max_, MPO_mean(1–4)_ and the absolute MPO_mean(1–8)_ (Table 3). In RSH-VHL, MPO_mean(5–8)_ (both relative and absolute, *p* < 0.01) and the relative MPO_mean(1–8)_ (*p* = 0.01) were higher whereas S_dec_ was lower (*p* = 0.01) at Post-1 than at Pre- (time effect for the three parameters). These parameters were not different between Post-2 and Pre- (*p* = 0.14–0.91) and between Post-1 and Post-2 (*p* = 0.11–0.35). Furthermore, RPE of the RSA test in RSH-VHL was lower at Post-1 than at both Pre- and Post-2 (*p* < 0.01) and not different between Pre- and Post-2 (*p* = 0.44). In RSN, no change was observed for any parameter. The comparison between RSH-VHL and RSN shows that the absolute MPO_mean(1–5)_ (*p* = 0.59–0.85), the relative MPO_mean(1–8)_ (*p* = 0.1–0.54), and S_dec_ (*p* = 0.09–0.52) were not different between conditions whatever the testing session. On the other hand, the relative MPO_mean(5–8)_ was higher (*p* = 0.04) and RPE was lower (*p* = 0.03) in RSH-VHL than in RSN at Post-1 and not different between conditions at Pre- (*p* = 0.92 and 0.46) and Post-2 (*p* = 0.28 and 0.42).

The repetition-by-repetition analysis throughout the RSA test shows that in RSH-VHL, the mean power output (both absolute and relative) was greater at Post-1 than at Pre-from the 6^th^ to the 8^th^ repetition and greater at Post-1 than at Post-2 in the 8^th^ repetition (absolute values) and the 7^th^ and 8^th^ repetition (relative values) (Figure 2). No difference was observed in the RSN group.

#### 3.2.2 Perceptual-motor capacity

There was no significant difference between groups in the number of hits in any of the four tests nor in the mean number of hits per test, whatever the testing session (Table 3). In both groups, the number of hits was not different between testing sessions in tests 1, 2 and 3 and was higher both at Post-1 and Post-2 than at Pre-in test 4.

#### 3.2.3 Cardiorespiratory parameters

During the 25-s repetitions, the ANOVA shows a time effect for $\dot{V}O_{2}$ , HR and $\dot{V}O_{2}$ /HR (Table 4). However, no difference was found in these parameters between testing sessions within groups. For all the other parameters ( $\dot{V}E$ , PETO_2_, PETCO_2_ and SpO_2_) the ANOVA did not reveal any significant effect.

**TABLE 4:** Mean cardio-respiratory parameters before (Pre-), 5 days after (Post-1), and 12 days after (Post-2) the training period.

	RSH-VHL			RSN			ANOVA P-value		
	Pre-	Post-1	Post-2	Pre-	Post-1	Post-2	T	C	T × C
25-s repetitions									
$\dot{V}O_{2}$ (L.min^-1^)	1.34 ± 0.3	1.63 ± 0.4	1.51 ± 0.4	1.30 ± 0.2	1.47 ± 0.7	1.38 ± 0.6	**0.04**	0.56	0.78
HR (bpm)	170 ± 7	166 ± 7	166 ± 9	171 ± 9	168 ± 11	168 ± 10	**0.03**	0.70	0.91
$\dot{V}O_{2}$ /HR (mL.beat^-1^)	7.9 ± 1.8	9.8 ± 2.7	9.1 ± 2.5	7.6 ± 1.2	8.8 ± 4.6	8.2 ± 4.1	**0.02**	0.57	0.79
$\dot{V}E$ (L.min^-1^)	111 ± 21	114 ± 20	118 ± 21	108 ± 22	120 ± 22	117 ± 29	0.12	0.94	0.54
PETO_2_ (mmHg)	121 ± 3	121 ± 3	120 ± 3	122 ± 4	123 ± 3	121 ± 2	0.09	0.23	0.59
PETCO_2_ (mmHg)	28.9 ± 2.6	29.8 ± 2.9	29.7 ± 4.5	28.6 ± 2.5	29.9 ± 2.7	29.7 ± 2.9	0.11	0.96	0.92
SpO_2_ (%)	96.4 ± 1.2	96.5 ± 0.6	96.5 ± 0.7	96.8 ± 1.0	96.6 ± 0.9	96.5 ± 0.9	0.78	0.66	0.44
25-s recovery periods									
$\dot{V}O_{2}$ (L.min^-1^)	1.57 ± 0.4	1.79 ± 0.5	1.74 ± 0.4	1.51 ± 0.4	1.66 ± 0.5	1.61 ± 0.5	0.06	0.57	0.93
HR (Bpm)	173 ± 6	168 ± 7*	169 ± 8	174 ± 10	173 ± 11	172 ± 10	**0.04**	0.47	0.41
$\dot{V}O_{2}$ /HR (mL.beat^-1^)	9.1 ± 2.3	10.7 ± 3.0*	10.3 ± 2.4	8.8 ± 2.8	9.7 ± 3.4	9.5 ± 3.5	**0.01**	0.56	0.77
$\dot{V}E$ (L.min^-1^)	99 ± 20	101 ± 19	102 ± 19	93 ± 20	103 ± 14	98 ± 21	0.12	0.70	0.34
PETO_2_ (mmHg)	114 ± 3	115 ± 3	114 ± 3	113 ± 5	114 ± 4	113 ± 4	0.09	0.50	0.98
PETCO_2_ (mmHg)	33.7 ± 2.9	34.6 ± 2.8	35.1 ± 3.7	34.3 ± 4.4	35.2 ± 3.5	35.2 ± 3.5	0.10	0.77	0.86
SpO_2_ (%)	97.4 ± 0.7	97.4 ± 0.5	97.5 ± 0.6	97.8 ± 0.9	97.6 ± 0.7	97.5 ± 0.8	0.25	0.54	0.24

Values are mean ± SD.

RSH-VHL, repeated-sprint training in hypoxia induced by voluntary hypoventilation at low lung volume; RSN, repeated sprint training with unrestricted breathing; $\dot{V}O_{2,}\text{ oxygen uptake}$ ; HR, heart rate; $\dot{V}O_{2}\;/\text{HR},\text{oxygen pulse};$ $\dot{V}E$ , expired ventilation; PETO_2_, end-tidal partial pressure in oxygen; PETCO_2,_ end-tidal partial pressure in carbon dioxide; SpO_2_, arterial oxygen saturation; T, time effect; C, condition effect; T × C, interaction effect (time × condition); bold values show significant difference. *, significantly different from Pre-within group; *p* < 0.05.

During the 25-s recovery periods, HR was lower and $\dot{V}O_{2}$ /HR higher at Post-1 than at Pre-in RSH-VHL (both *p* = 0.04) but not different between Post-1 and Post-2 (*p* = 0.92 and 0.90) and between Pre- and Post-2 (*p* = 0.17 and 0.15). There was no change in these parameters between testing sessions in RSN. For all the other parameters, there was no time, condition or interaction effect.

**FIGURE 3:**
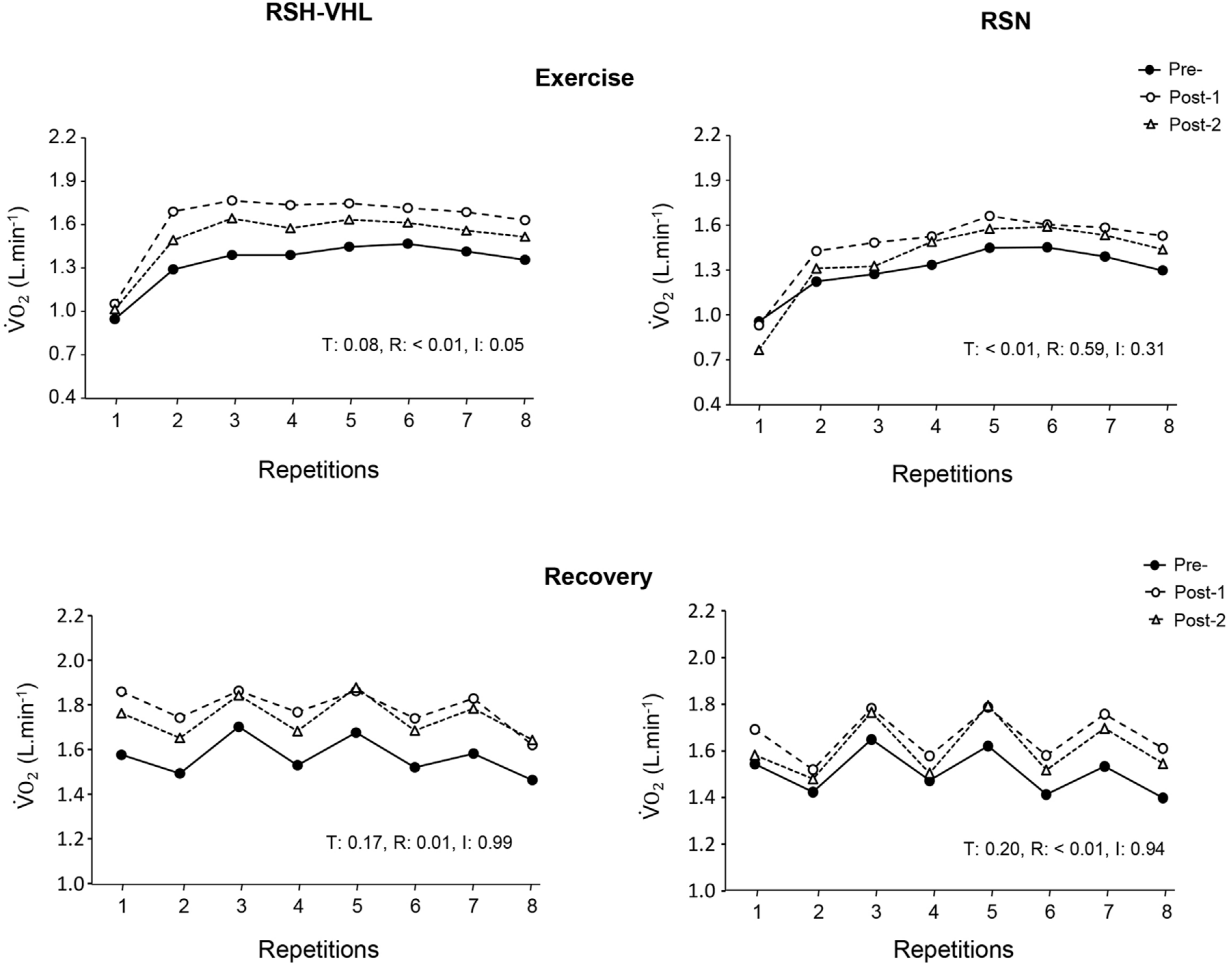
Oxygen uptake ( $\dot{V}O_{2}$ ) measured in each of the 25-s repetitions (upper panels) and recovery periods (lower panels) of the repeated-sprint ability test before (Pre-), 5 days after (Post-1) and 12 days after (Post-2) the repeated-sprint training with voluntary hypoventilation at low lung volume (RSH-VHL, left panels) and with unrestricted breathing (RSN, right panels). T: ANOVA time effect (Pre-vs. Post-1 vs. Post-2); R: ANOVA repetition effect; I: ANOVA interaction effect. Standard deviation is not included for more clarity.

The repetition-by-repetition analysis for $\dot{V}O_{2}$ did not reveal any significant difference between testing sessions both in RSH-VHL and in RSN (Figure 3).

#### 3.2.4 Near-infrared spectroscopy

**FIGURE 4:**
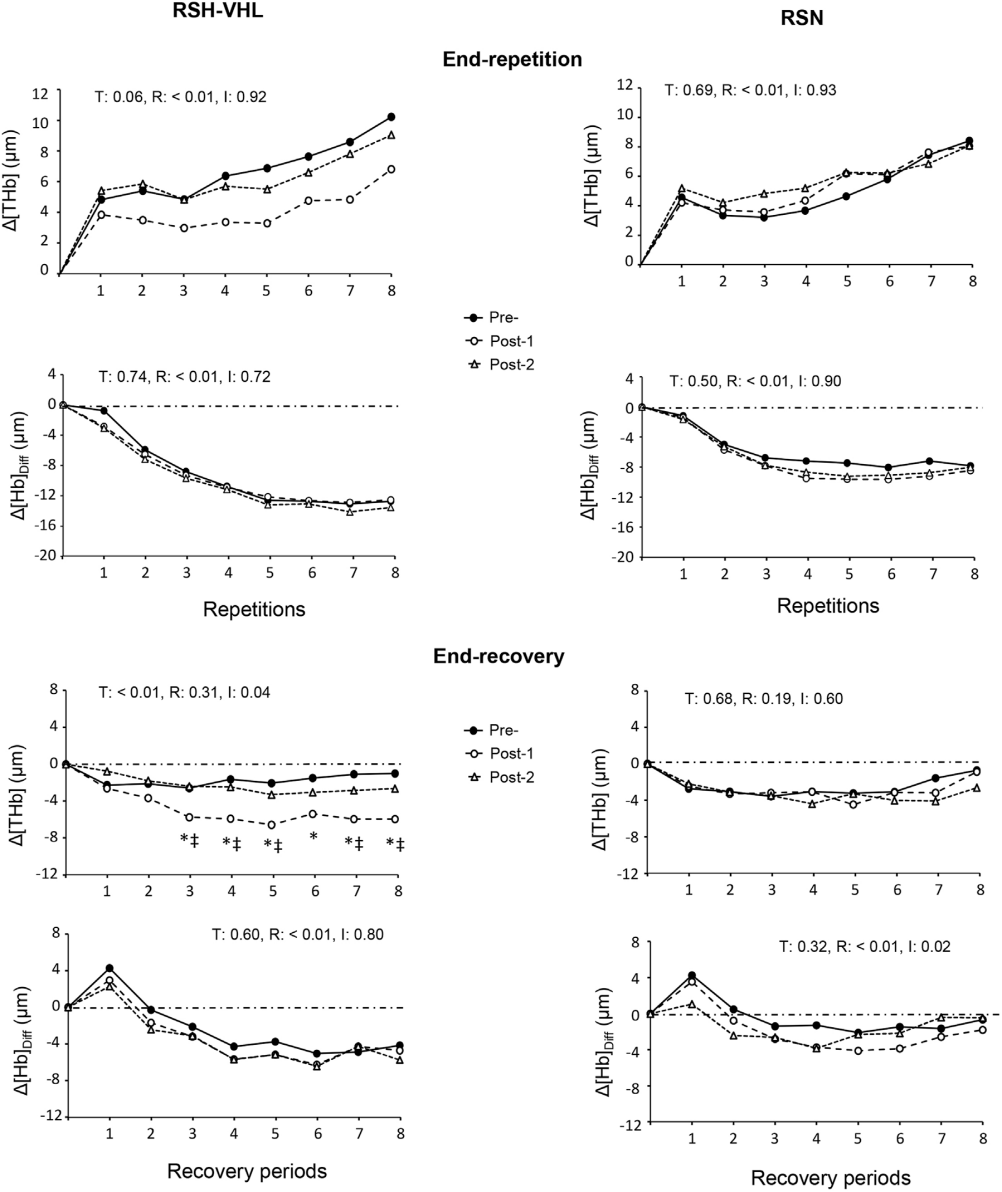
Changes in the mean cerebral total haemoglobin concentration (Δ[THb]) and cerebral oxygenation (Δ[Hb]_Diff_) at the end of each repetition (upper four panels) and recovery period (lower four panels) of the repeated-sprint ability test before (Pre-), 5 days after (Post-1) and 12 days after (Post-2) the repeated-sprint training with voluntary hypoventilation at low lung volume (RSH-VHL, left panels) and with unrestricted breathing (RSN, right panels). T: ANOVA time effect (Pre-vs. Post-1 vs. Post-2); R: ANOVA repetition effect; I: ANOVA interaction effect. *, significant difference with the same sprint at Pre; ^‡^, significant difference with the same sprint at Post-2; *p* < 0.05. Standard deviation is not included for more clarity.

At the cerebral level, there was no time, condition or interaction effect for the mean Δ[THb] and Δ[Hb]_diff_ at the end of the 25-s repetitions nor for the mean Δ[Hb]_diff_ at the end of the recovery periods (Table 5). On the other hand, in RSH-VHL, the mean end-recovery Δ[THb] was lower at Post-1 than at both Pre- and Post-2 (*p* < 0.01) whereas there was no change in the RSN group. The repetition-by-repetition analysis shows that in RSH-VHL, the end-recovery Δ[THb] was lower at Post-1 than at Pre- and Post-2 from the 3^rd^ to the 8^th^ repetition (Figure 4). With regard to the end-recovery Δ[Hb]_diff_ and the end-repetition Δ[THb] and Δ[Hb]_diff_, no time or interaction effect was found in either group.

**TABLE 5:** Mean NIRS values before (Pre-), 5 days after (Post-1), and 12 days after (Post-2) the training period.

	RSH-VHL			RSN			ANOVA *P*-value		
	Pre-	Post-1	Post-2	Pre-	Post-1	Post-2	T	C	T × C
Cerebral									
End-repetition									
Δ[THb] (µM)	6.8 ± 4.5	4.2 ± 6.1	6.3 ± 5.5	5.1 ± 4.8	5.5 ± 4.7	5.9 ± 5.3	0.13	0.89	0.10
Δ[Hb]_Diff_ (µM)	−9.7 ± 2.8	−10.0 ± 4.1	−10.6 ± 3.5	−6.3 ± 6.2	−7.7 ± 5.0	−7.7 ± 5.2	0.42	0.13	0.85
End-recovery									
Δ[THb] (µM)	−1.8 ± 4.1	−5.2 ± 3.8*^‡^	−2.4 ± 2.9	−2.6 ± 3.4	−2.9 ± 2.1	−3.4 ± 1.9	**0.02**	0.91	**0.02**
Δ[Hb]_Diff_ (µM)	−2.5 ± 2.9	−3.5 ± 3.6	−3.8 ± 4.3	−0.5 ± 5.2	−2.0 ± 4.0	−2.3 ± 6.2	0.21	0.34	0.94
Muscle									
End-repetition									
Δ[THb/Mb] (µM)	−5.0 ± 7.5	−1.5 ± 7.6	−5.1 ± 5.3	−3.5 ± 6.2	−2.4 ± 6.8	−4.9 ± 6.1	0.09	0.92	0.70
Δ[Hb/Mb]_Diff_ (µM)	−31.0 ± 18	−41.9 ± 29	−38.3 ± 19	−29.2 ± 17	−38.1 ± 21	−38.4 ± 19	**< 0.01**	0.86	0.81
End-recovery									
Δ[THb/Mb] (µM)	0.4 ± 10	4.7 ± 11*^a^	0.2 ± 10	3.0 ± 7.8	2.6 ± 8.3	1.5 ± 8.1	**0.01**	0.89	**0.03**
Δ[Hb/Mb]_Diff_ (µM)	−7.4 ± 7.0	−14.9 ± 16	−13.0 ± 6.1	−8.3 ± 9.0	−15.2 ± 12.1	−14.7 ± 11	**0.01**	0.81	0.96
End-repetition-to-end-recovery reoxygenation									
Reoxy [THb/Mb] (µM)	5.5 ± 4.5	6.1 ± 4.5	5.3 ± 7.6	6.6 ± 4.2	5.0 ± 3.6	6.5 ± 8.0	0.93	0.87	0.56
Reoxy [Hb/Mb]_Diff_ (µM)	23.6 ± 14	26.9 ± 16	25.3 ± 17	20.9 ± 10	22.9 ± 13	23.7 ± 12	0.65	0.40	0.84

Values are mean ± SD.

NIRS, near-infrared spectroscopy; RSH-VHL, repeated-sprint training in hypoxia induced by voluntary hypoventilation at low lung volume; RSN, repeated-sprint training with normal breathing; Δ[THb], change in total haemoglobin concentration; Δ[Hb]_Diff_, change in haemoglobin oxygenation; Δ[THb/Mb], change in total haemoglobin/myoglobin concentration; Δ[Hb/Mb]_Diff_, change in haemoglobin/myoglobin oxygenation; Reoxy [THb/Mb] (µM), change in total haemoglobin/myoglobin concentration during the recovery periods; Reoxy [Hb/Mb]_Diff_, haemoglobin/myoglobin reoxygenation. T, time effect; C, condition effect; T × C, interaction effect (time × condition); bold values show significant difference. *, significantly different from Pre-within group; ^a^ significantly different from Post-2 within group; *p* < 0.05.

**FIGURE 5:**
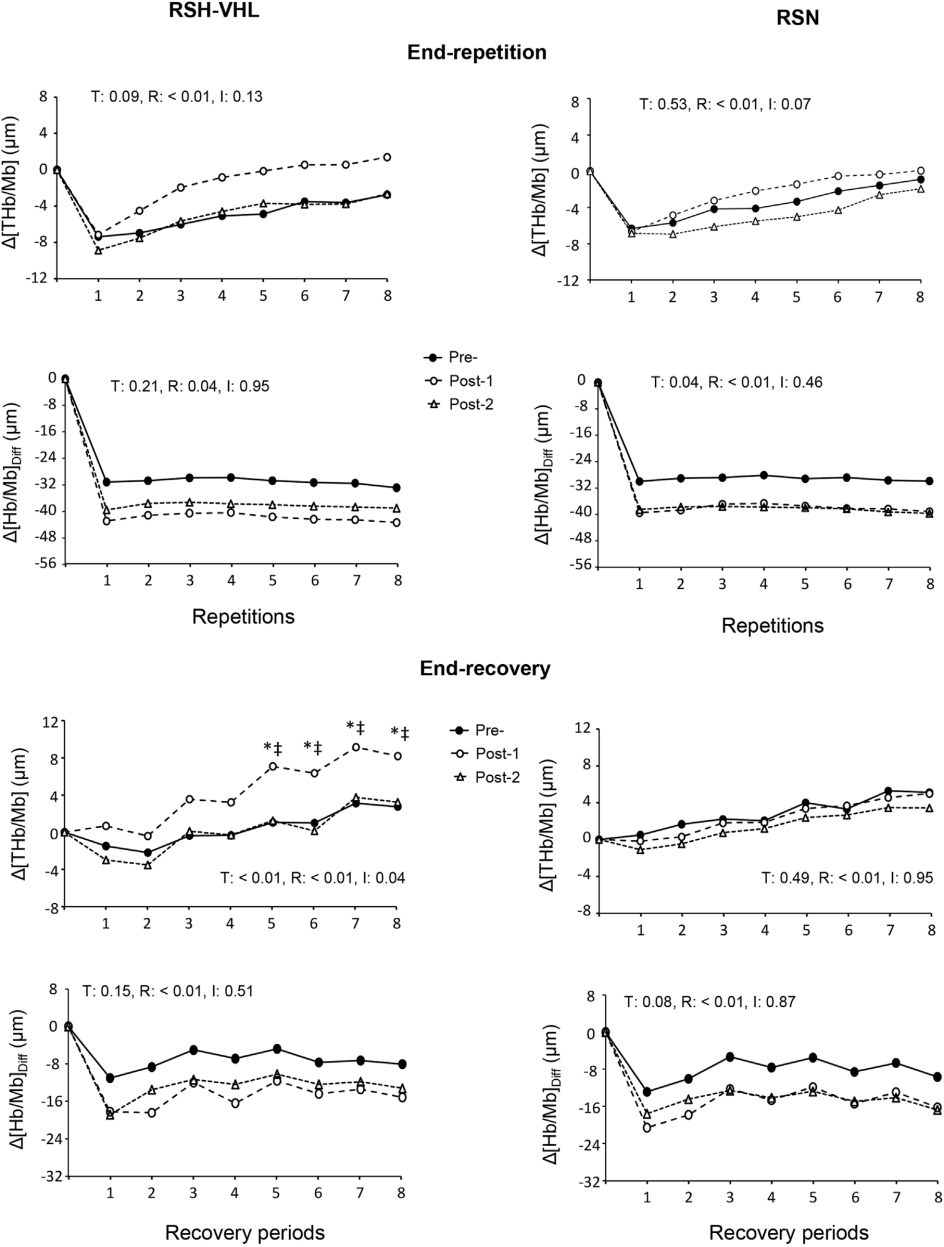
Changes in the mean muscle total haemoglobin/myoglobin concentration (Δ[THb/Mb] and muscle oxygenation (Δ[Hb/Mb]_Diff_ at the end of each repetition (upper four panels) and recovery period (lower four panels) of the repeated-sprint ability test before (Pre-), 5 days after (Post-1) and 12 days after (Post-2) the repeated-sprint training with voluntary hypoventilation at low lung volume (RSH-VHL, left panels) and with unrestricted breathing (RSN, right panels). T: ANOVA time effect (Pre vs. Post-1 vs. Post-2); R: ANOVA repetition effect; I: ANOVA interaction effect. *, significant difference with the same sprint at Pre-; ^‡^, significant difference with the same sprint at Post-2; *p* < 0.05. Standard deviation is not included for more clarity.

At the muscle level, despite a time effect for the mean Δ[Hb/Mb]_Diff_ both at the end of the repetitions and recovery periods, the post-hoc analysis did not reveal any significant difference between testing sessions within groups (RSH-VHL: *p* = 0.09–0.95; RSN: *p* = 0.08–0.97). The ANOVA did not show any significant effect for the mean end-repetition Δ[THb/Mb] as well as for the mean Reoxy [THb/Mb] and Reoxy [Hb/Mb]_Diff_. On the other hand, in RSH-VHL, the mean Δ[THb/Mb] at the end of the recovery periods was higher at Post-1 than at both Pre- and Post-2 (*p* < 0.01) whereas no change was found in the RSN group (*p* = 0.75–0.98). The repetition-by-repetition analysis shows that in RSH-VHL, the end-recovery Δ[THb/Mb] was higher at Post-1 than at Pre- and Post-2 from the 5^th^ to the 8^th^ repetition (Figure 5). With regard to the end-recovery Δ[Hb/Mb]_diff_ and the end-repetition Δ[THb/Mb] and Δ[Hb/Mb]_diff_, no time or interaction effect was found in both groups or if so (time effect for end-repetition Δ[Hb/Mb]_diff_ in RSN only), no difference was found between testing sessions whatever the repetition number (Figure 5).

## 4 Discussion

This study is the first to investigate the effects of a repeated-sprint training in hypoxia induced by voluntary hypoventilation at low lung volume including end-expiratory breath holding of maximal duration. The results show that elite judo athletes who followed this training regimen for 4 weeks significantly improved their ability to repeat “all-out” efforts (lower fatigue, greater performance in the second part of the test) unlike the judokas who performed the same training with unrestricted breathing. Along with the improved RSA, we report for the first time an increase in muscle blood volume and a concomitant decrease in cerebral blood volume during the recovery periods of a repeated-sprint exercise after the RSH-VHL intervention.

### 4.1 Performance

Previous studies have shown the interest of performing a high-intensity VHL training to improve RSA in athletes of different sporting disciplines. After 2 weeks (6 sessions), 4 weeks (7 sessions) and 6 weeks (12 sessions) of RSH-VHL, the maximum number of sprints until exhaustion (open-loop RSA test design) was significantly increased in highly-trained swimmers (+35%), male rugby players (+64%) and female soccer players (+17%), respectively (Ait Ali Braham et al., 2024; Fornasier Santos et al., 2018; Trincat et al., 2017). In a closed-loop testing design (i.e., with a fixed number of sprints), the mean power output and the mean velocity were increased by 8% and 4% in cyclists and ice hockey players after 3 and 5 weeks of RSH-VHL, respectively (Brocherie et al., 2022; Woorons et al., 2019a). In these latter studies, the performance improvement was accompanied by a 4% reduction in fatigue over the RSA test. Lower fatigue during a RSA test has also been reported in basketball players and soccer players who performed four to 6 weeks (8–12 sessions) of RSH-VHL (Ait Ali Braham et al., 2024; Lapointe et al., 2020). Remarkably, in all these studies, the greater increase in performance was observed only or mainly in the second part of the test. In the present study, the 3.5% reduction in fatigue and the higher power output in the last three repetitions of the RSA test at Post-1 in the RSH-VHL group confirms the aforementioned findings. However, although the use of maximal EEBH induced a much stronger hypoxic stimulus than previously reported, the RSA improvement does not appear to be of greater magnitude. Yet it is important to note that the participants of the present study, unlike the previous ones, were elite athletes who generally have less room for performance improvement. Furthermore, this study shows, for the first time, a superiority of the RSH-VHL intervention over the RSN one to increase performance in the second half of a RSA test. Therefore, our results demonstrate that performance can also be enhanced in high-level athletes when using the latest approach of the VHL method.

### 4.2 Muscle near-infrared spectroscopy

The increase in muscle Δ[THb/Mb] during the recovery periods of the RSA test after RSH-VHL represents a new and important finding. These changes, which reveal greater muscle blood volume, were observed from the 5^th^ to the 8^th^ sprint at Post-1 (Figure 5) and were therefore concomitant with the improvement in performance (Figure 2). In previous studies, no change in muscle Δ[THb/Mb] was reported after 3–4 weeks of RSH-VHL (Lapointe et al., 2020; Woorons et al., 2019a). The main reason is probably that in these studies, unlike the present one, the EEBH were not maximal which did not induce a comparable fall in SpO_2_ (mean values: 87.7% vs. 78.7%) and therefore a similar hypoxic stimulus. In a recent (acute) study, it was shown that the repetition of supramaximal running exercises with EEBH up to the breaking point could decrease SpO_2_ down to ∼75% and induce a large and early increase in muscle blood volume as compared to the same exercise with unrestricted breathing (Woorons et al., 2023). Conversely, the repetition of sprints with non-maximal EEBH (i.e., 6-s duration) did not yield any change in muscle Δ[THb/Mb] (Woorons et al., 2017; Woorons et al., 2019b). As suggested (Woorons et al., 2023), the strong hypoxic effect induced by the maximal EEBH may (acutely) provoke a compensatory vasodilation, possibly mediated by the release in nitric oxide (Casey et al., 2010; Wilkins et al., 2008), to preserve muscle O_2_ delivery. The hypoxia-induced muscle vasodilation in this high-intensity exercise condition is probably reinforced by the strong hypercapnic effect through a large increase in hydrogen ions (H^+^). In the present study, we suggest that the repetition of this phenomenon over the 4-week training period may have induced physiological adaptations leading to improved muscle perfusion observed during the RSA test. Among the factors possibly involved in this physiological change, greater capillary-to-fiber ratio, fiber cross-sectional area, and/or myoglobin content seem to be the best candidates.

An enhanced muscle perfusion during the recovery periods of a repeated-sprint exercise provides greater oxygen availability which is paramount for improving RSA (Girard et al., 2011). Indeed, it may increase the resynthesis of phosphocreatine, which is an important energy component in repeated sprints, as well as the removal of waste metabolites (e.g., inorganic phosphate, H^+^). This may explain, at least in part, the RSA improvement in the RSH-VHL group at Post-1. The fact that the increase in muscle Δ[THb/Mb] occurred from the second half of the RSA test may be due to the presence of numerous vasodilator metabolites, in particular H^+^ since high-intensity VHL training has been shown to increase the anaerobic glycolysis activity (Trincat et al., 2017; Woorons et al., 2016; Woorons et al., 2019a). In the same time, the elimination of these waste metabolites via the oxygen pathway probably became determinant at this point of the test for maintaining a high level of performance. Thus, the greater muscle perfusion in the recovery period following the 5^th^ sprint of the RSA test should have had a positive impact on performance of the 6^th^ sprint, and so on until the 8^th^ sprint.

It is remarkable that, in the previous RSH-VHL studies, RSA performance was increased without any change in muscle Δ[THb/Mb]. However, in these studies, higher oxygen uptake (Woorons et al., 2019a) or greater muscle reoxygenation (Lapointe et al., 2020) were reported during the recovery periods of the RSA test. Such physiological changes were not observed in the present study, nor was there any improvement in muscle oxygen utilization (i.e., unchanged Δ[Hb/Mb]_Diff_ despite a tendency to a decrease in both groups), contrary to our hypothesis. Therefore, based on the present and previous findings, we can suggest that the physiological adaptations induced by RSH-VHL may be of a different nature depending on the degree of hypoxia (and probably hypercapnia) induced by EEBH and, possibly, on the mode of exercise and the muscle mass involved.

### 4.3 Cerebral near-infrared spectroscopy

The decrease in cerebral Δ[THb] that also occurred in the recovery periods of the RSA test at Post-1 in the RSH-VHL group represents another interesting and original finding. This result, which indicates lower regional cerebral blood volume and which was accompanied by an unchanged cerebral Δ[Hb]_diff_, is surprising and contradicts our hypothesis. In their acute study, Woorons et al. (2023) showed that the use of maximal EEBH during supramaximal exercise induced a dramatic increase in cerebral Δ[THb] and a (fairly moderate) decrease in cerebral Δ[Hb]_diff_. Like what happened at the muscle level, one could have expected that the repetition of such an exercise condition over several weeks of training would have induced physiological adaptations leading to improved cerebral oxygenation. The fact that it was not so weakens the possibility that the RSA gains after RSH-VHL could be due, in part, to lower central fatigue (Woorons, 2014). The decrease in cerebral Δ[THb] after RSH-VHL may, however, not represent a deterioration in the brain’s oxygenation capacity. Indeed, to cope with the high muscle perfusion during the recovery periods of the RSA test without any increase in $\dot{V}O_{2}$ , and probably in cardiac output, we postulate that a blood volume redistribution may have occurred after the RSH-VHL intervention. If so, other organs than the brain may have been involved. With this regard, considering that the NIRS data do not distinguish between arterial, capillary and venous blood, it may be possible that the cerebral Δ[THb] measures may have been affected by blood flow in extracerebral and superficial tissues, even though the NIRS measurements have been shown to be minimally affected by skin blood flow (Mancini et al., 1994). In any case, it is highly unlikely that the lower cerebral Δ[THb] was due to a cerebral vasoconstriction resulting from hyperventilation-induced hypocapnia given that both $\dot{V}E$ and PETCO_2_ remained unchanged after RSH-VHL. It is also unlikely that cerebral blood flow was negatively affected by the reduced cerebral Δ[THb] due to the brain autoregulation to maintain its adequate perfusion (Ogoh and Ainslie, 2009).

### 4.4 Oxygen uptake and heart rate

The hypothesis of a blood volume redistribution in response to the increased muscle perfusion during the recovery periods of the RSA test after RSH-VHL would be reinforced if $\dot{V}O_{2}$ and cardiac output were at their maximum (Joyner and Casey, 2015). Yet, the $\dot{V}O_{2}$ values were surprisingly low, suggesting that there was room for an increase in cardiac output to insure adequate perfusion of all organs. However, the aerobic power of elite judo athletes has been shown to be quite low and not different from that of non-elite judokas (Franchini et al., 2011). Typical maximal $\dot{V}O_{2}$ of 50–55 mL kg^−1^ min^−1^ and 40–45 mL kg^−1^ min^−1^ have been reported for male and female judo athletes, respectively, which is close to what is generally found in recreational active subjects. Besides, the $\dot{V}O_{2}$ values of the present study are in line with what has been reported during 20-s repeated rowing sprints in recreational active male and female subjects (Clausen and Astorino, 2024). Although the RSH-VHL intervention did not increase $\dot{V}O_{2}$ , it induced lower HR and higher $\dot{V}O_{2}$ /HR in the recovery periods of the RSA test at Post-1. This outcome confirms the greater $\dot{V}O_{2}$ /HR reported after 3 weeks of RSH-VHL in cycling (Woorons et al., 2019a). It supports the assumption that this kind of training could increase stroke volume when it includes EEBH of maximal duration (Woorons et al., 2021a), even though the use of $\dot{V}O_{2}$ /HR as indicator of stroke volume during repeated-sprint exercise can be questioned.

### 4.5 Perceptual motor capacity

Another original aim of this study was to assess the effects of RSH-VHL on perceptual-motor capacity. The fact that cerebral oxygenation was not improved after this training intervention may explain why performance in the perceptual-motor tests was mainly not augmented or if so (4^th^ test after the last sprint of the RSA test), not to a greater extent than in the RSN group. The role of an adequate cerebral oxygenation on cognitive performance has been previously demonstrated (Dupuy et al., 2015). Furthermore, it has been shown that the reaction time in executive cognitive tests close to those used in this study was significantly related to prefrontal cerebral oxygenation (Mekari et al., 2015). The better performance observed in both groups during the 4^th^ perceptual-motor test at Post-1 and Post-2 may be due to a learning effect, the influence of which could have been more pronounced when fatigue was at its peak. Although RSH-VHL does not appear to ameliorate perceptual-motor ability, further studies using other types of perceptual-motor tests are needed before drawing any conclusions. As for the perceived response to exercise, it is noteworthy that RPE of the RSA test decreased at Post-1 in the RSH-VHL group and was lower than in RSN. Lower RPE in a closed-loop RSA test has already been reported after RSH-VHL (Lapointe et al., 2020). We postulate that the enhanced recovery induced by higher muscle perfusion in the present study, or greater muscle reoxygenation in the previous one (Lapointe et al., 2020), delayed fatigue so that subjects were not completely exhausted at the end of these specific closed-loop tests. In this case, they would probably have been capable of performing one or a few more sprints before reaching the same RPE as in the Pre-test.

### 4.6 Practical applications

The results of the present study may have interesting practical applications not only for judo athletes or, more generally, for combat sports but also for athletes engaged in other intermittent sporting disciplines (e.g., team sports, racket sports, cycling). They show that performing a period of RSH-VHL including maximal EEBH can optimize the recovery periods during repeated high-intensity efforts and, consequently, allow to maintain a high level of performance, in particular in the second part of a match or combat when fatigue is more pronounced. In this study, the physiological adaptations and the increase in performance were observed 5 days after the last training session but were no longer visible 12 days after. This provides indications for the periodization of RSH-VHL before a competition. However, the results obtained in Post-2 must be interpreted with caution. Indeed, the rowing exercises were used by the participants exclusively for the purpose of this study and were stopped after the last training session. Therefore, a detraining effect probably occurred after a certain period of time, which may not have been the case in athletes using their usual mode of exercise. Furthermore, in the context of physical preparation, we suggest to maintain one weekly session of RSH-VHL after performing a period of such training so that the physiological benefits could possibly be maintained over several weeks.

### 4.7 Limitations

A first limitation of this study is the lack of blood measurements during the RSA tests. Taking blood samples, in particular in the second part of the test, would have been useful especially for analysing pH and lactate concentration. This would have allowed to test the hypothesis of greater H^+^ elimination, and more generally of waste metabolites, during the recovery periods of the RSA test after RSH-VHL. Blood measurements would have also enabled to determine the concentration of potassium ions which was found to be lower and linked to improved performance after 4 weeks of RSH-VHL in basketball players (Lapointe et al., 2020). Another limitation is that cerebral and muscle blood flow were not measured either. A possible decrease or maintenance in the former and increase in the latter at Post-1 of the RSH-VHL intervention could therefore not be ascertained. However, the techniques used for these measures are somewhat difficult to implement during repeated-sprint exercises. On the other hand, the use of the NIRS technique was probably relevant since it allows continuous recording and provides data that have quite similar time course as cerebral blood flow when measured by transcranial doppler (Hirth et al., 1997). Yet, it is important to remind that the change in [O_2_Hb] and [HHb] are somewhat affected by blood flow through extracerebral and superficial tissues. In addition, one limitation regarding the use of the NIRS technique is that we did not measure the tissue oxygenation index, which provides absolute values (expressed in %) and probes oxygenation in deeper tissues when combined with spatially resolved spectroscopy. Finally, like in all studies using breath control, participants could not be blinded to the RSH-VHL intervention. Consequently, the RSA gains might be attributed, at least in part, to a placebo effect. However, the acute and chronic effects of the VHL approach have now been well investigated and the RSA improvement reported in the present study may be explained mainly by physiological rather than psychological factors. In addition, the role of a placebo effect to explain the RSA improvement after high-intensity VHL training has been recently challenged (Woorons et al., 2020).

## 5 Conclusion

Four weeks of RSH-VHL including EEBH of maximal duration improved the ability of elite judo athletes to repeat high-intensity efforts. Our findings suggest that this improvement was made possible through an enhanced muscle perfusion and consequently higher oxygen supply during the recovery periods of the RSA test. In the same time, $\dot{V}O_{2}$ remained unchanged whereas regional cerebral blood volume decreased. These outcomes tend to show that a blood volume redistribution occurred after RSH-VHL to meet the large increase in muscle perfusion. The latest approach of the VHL method may be of interest for a large number of athletes involved in different intermittent sports to improve their performance.

## Data Availability

The raw data supporting the conclusions of this article will be made available by the authors, without undue reservation.

## References

[B1] Ait Ali Braham M. Ouchene Y. Woorons X. (2024). Effects of a 6-week repeated-sprint training with voluntary hypoventilation at low and high lung volume on repeated-sprint ability in female soccer players. Int. J. Sports Physiol. Perf. 19 (5), 463–470. 10.1123/ijspp.2023-0392 38412852

[B2] Billaut F. Buchheit M. (2013). Repeated-sprint performance and vastus lateralis oxygenation: effect of limited O availability. Scand. J. Med. Sci. Sports. 23, e185–193. 10.1111/sms.12052 23362832

[B3] Billaut F. Kerris J. P. Rodriguez R. F. Martin D. T. Gore C. J. Bishop D. J. (2013). Interaction of central and peripheral factors during repeated sprints at different levels of arterial O2 saturation. PLoS One, 8, e77297. 10.1371/journal.pone.0077297 24155938 PMC3796493

[B4] Boushel R. Piantadosi C. A. (2000). Near-infrared spectroscopy for monitoring muscle oxygenation. Acta Physiol. Scand. 168, 615–622. 10.1046/j.1365-201x.2000.00713.x 10759598

[B5] Brocherie F. Cantamessi G. Millet G. P. Woorons X. (2022). Effects of repeated-sprint training in hypoxia induced by voluntary hypoventilation on performance during ice hockey off-season. Int. J. Sports Sci. Coaching. 18, 446–452. 10.1177/17479541221079531

[B6] Casey D. P. Madery B. D. Curry T. B. Eisenach J. H. Wilkins B. W. Joyner M. J. (2010). Nitric oxide contributes to the augmented vasodilatation during hypoxic exercise. J. Physiol. 588, 373–385. 10.1113/jphysiol.2009.180489 19948661 PMC2821731

[B7] Clausen R. D. Astorino T. A. (2024). Excess post-exercise oxygen consumption after reduced exertion high-intensity interval training on the cycle ergometer and rowing ergometer. Eur. J. Appl. Physiol. 124, 815–825. 10.1007/s00421-023-05309-x 37787925

[B8] Duncan A. Meek J. H. Clemence M. Elwell C. E. Fallon P. Tyszczuk L. (1996). Measurement of cranial optical path length as a function of age using phase resolved near infrared spectroscopy. Pediatric Research, 39, 889–894. 10.1203/00006450-199605000-00025 8726247

[B9] Dupuy O. Gauthier C. J. Fraser S. A. Desjardins-Crèpeau L. Desjardins M. Mekary S. (2015). Higher levels of cardiovascular fitness are associated with better executive function and prefrontal oxygenation in younger and older women. Front. Hum. Neurosci. 9, 66. 10.3389/fnhum.2015.00066 25741267 PMC4332308

[B10] Fornasier-Santos C. Millet G. P. Woorons X. (2018). Repeated-sprint training in hypoxia induced by voluntary hypoventilation improves running repeated-sprint ability in rugby players. Eur. J. Sport Sci. 18, 504–512. 10.1080/17461391.2018.1431312 29400616

[B11] Franchini E. Del Vecchio F. B. Matsushigue K. A. Artioli G. G. (2011). Physiological profiles of elite judo athletes. Sports Med. 41, 147-166. 10.2165/11538580-000000000-00000 21244106

[B12] Girard O. Brocherie F. Goods P. S. R. Millet G. P. (2020). An Updated Panorama of “Living Low-Training High” Altitude/Hypoxic Methods. Living. 2, 26. 10.3389/fspor.2020.00026 PMC7739748 33345020

[B13] Girard O. Brocherie F. Millet G. P. (2017). Effects of altitude/hypoxia on single- and multiple-sprint performance: a comprehensive review. Sports Med. 47, 1931–1949. 10.1007/s40279-017-0733-z 28451905

[B14] Girard O. Mendez-Villanueva A. Bishop D. (2011). Repeated-sprint ability - part I: factors contributing to fatigue. Sports Med. 41, 673–694. 10.2165/11590550-000000000-00000 21780851

[B15] Glaister M. Howatson G. Pattison J. R. McInnes G. (2008). The reliability and validity of fatigue measures during multiple-sprint work: an issue revisited. J. Strength Cond. Res. 22, 1597–1601. 10.1519/JSC.0b013e318181ab80 18714226

[B16] Grassi B. Pogliaghi S. Rampichini S. Quaresima V. Ferrari M. Marconi C. (2003). Muscle oxygenation and pulmonary gas exchange kinetics during cycling exercise on transitions in humans. J Appl. Physiol. 95, 149–158. 10.1152/japplphysiol.00695.2002 12611769

[B17] Hirth C. Obrig H. Valdueza J. Dirnagl U. Villringer A. (1997). Simultaneous assessment of cerebral oxygenation and hemodynamics during a motor task. A combined near infrared and transcranial Doppler sonography study. Adv. Exp. Med. Biol., 411, 461–469. 10.1007/978-1-4615-5865-1_59 9269463

[B18] Jobgen W. Meininger C. J. Jobgen S. C. Li P. Lee M. Smith S. B. (2009). Dietary L-Arginine supplementation reduces white fat gain and enhances skeletal muscle and brown fat masses in diet-induced obese rats. J. Nutr. 139, 230–237. 10.3945/jn.108.096362 19106310 PMC3151442

[B19] Joyner J. M. Casey D. P. (2013). Regulation of Increased Blood Flow (Hyperemia) to Muscles During Exercise: A Hierarchy of Competing Physiological Needs. Physiol. Rev. 95, 549–601. 10.1152/physrev.00035.2013 PMC4551211 25834232

[B20] Kume D. Akahoshi S. Yamagata T. Wakimoto T. Nagao N. (2016). Does voluntary hypoventilation during exercise impact EMG activity? Springerplus. 5, 149. 10.1186/s40064-016-1845-x 27026846 PMC4766162

[B21] Lapointe J. Paradis-Deschênes P. Woorons X. Lemaître F. Billaut F. (2020). Impact of Hypoventilation Training on Muscle Oxygenation, Myoelectrical Changes, Systemic [K^+^], and Repeated-Sprint Ability in Basketball Players. Living. 2, 29. 10.3389/fspor.2020.00029 PMC7739750 33345021

[B22] Lassala A. Bazer F. W. Cudd T. A. Datta S. Keisler D. H. Satterfield M. C. (2010). Parenteral administration of L-arginine prevents fetal growth restriction in undernourished ewes. J. Nutr. 140, 1242–1248. 10.3945/jn.110.125658 20505020 PMC2884328

[B23] MacDonald M. J. Tarnopolsky M. A. Green H. E. Hughson R. L. (1999). Comparison of femoral blood gases and muscle near-infrared spectroscopy at exercise onset in humans. J. Appl. Physiol. 86, 687-693. 10.1152/jappl.1999.86.2.687 9931209

[B24] Mancini D. M. Bolinger L. Li H. Kendric k K. Chance B. Wilson J.R. (1994). Validation of near-infrared spectroscopy in humans. J. Appl. Physiol. 77, 2740–2747. 10.1152/jappl.1994.77.6.2740 7896615

[B25] Mekari S. Fraser S. Bosquet L. Bonnéry C. Labelle V. Pouliot P. (2015). The relationship between exercise intensity, cerebral oxygenation and cognitive performance in young adults. Eur. J. Appl. Physiol. 115, 2189–2197. 10.1007/s00421-015-3199-4 26063061

[B26] Ogoh S. Ainslie P. N. (2009). Cerebral blood flow during exercise: mechanisms of regulation. J. Appl. Physiol. 107, 1370–1380. 10.1152/japplphysiol.00573.2009 19729591

[B27] Tan B. E. Yin Y. L. Kong X. F. Li P. Li X. Gao H. (2010). L-Arginine stimulates proliferation and prevents endotoxin-induced death of intestinal cells. Amino Acids. 38, 1227–1235. 10.1007/s00726-009-0334-8 19669080 PMC2850530

[B28] Toubekis A. G. Beidaris N. Botonis P. G. Koskolou M. (2017). Severe hypoxemia induced by prolonged expiration and reduced frequency breathing during submaximal swimming. J. Sports Sci. 35, 1025-1033. 10.1080/02640414.2016.1209304 27431779

[B29] Trincat L. Woorons X Millet G. P. (2017). Repeated-Sprint Training in Hypoxia Induced by Voluntary Hypoventilation in Swimming. Int. J. Sports Physiol. Perform. 12, 329–335. 10.1123/ijspp.2015-0674 27294771

[B30] van Beekvelt M. C. van Engelen B. G. Wevers R. A. Colier W. N. (2002). In vivo quantitative near-infrared spectroscopy in skeletal muscle during incremental isometric handgrip exercise. Clin. Physiol. Funct. Imaging. 22, 210–217. 10.1046/j.1475-097x.2002.00420.x 12076348

[B31] Wei J. Carroll R. J. Harden K. K. Wu G. (2012). Comparisons of treatment means when factors do not interact in two-factorial studies. Amino Acids. 42, 2031–2035. 10.1007/s00726-011-0924-0 21547361 PMC3199378

[B32] Whipp B.J. Higgenbotham M. B. Cobb F. C. (1996). Estimating exercise stroke volume from asymptotic oxygen pulse in humans. J. Appl. Physiol. 81, 2674–2679. 10.1152/jappl.1996.81.6.2674 9018521

[B33] Wilkins B. W. Pike T. L. Martin E. A. Curry T. B. Ceridon M. L. Joyner M. J. (2008). Exercise intensity-dependent contribution of beta-adrenergic receptor-mediated vasodilatation in hypoxic humans. J. Physiol. 586, 1195–1205. 10.1113/jphysiol.2007.144113 18048452 PMC2375634

[B34] Willis S. J. Alvarez L. Millet G. P. Borrani F. (2017). Changes in muscle and cerebral deoxygenation and perfusion during repeated sprints in hypoxia to exhaustion. Front. Physiol. 8, 846. 10.3389/fphys.2017.00846 29163193 PMC5671463

[B35] Woorons X. Mollard P. Pichon A. Duvallet A. Richalet J. P. Lamberto C. (2007). Prolonged expiration down to residual volume leads to severe arterial hypoxemia in athletes during submaximal exercise. Respir. Physiol. Neurobiol. 15, 75–82. 10.1016/j.resp.2007.02.017 17434347

[B36] Woorons X. Mollard P. Pichon A. Duvallet A. Richalet J.P. Lamberto C. (2008). Effects of a 4-week training with voluntary hypoventilation carried out at low pulmonary volumes. Respir. Physiol. Neurobiol. 160, 123–130. 10.1016/j.resp.2007.09.010 18160351

[B37] Woorons X. Bourdillon N. Vandewalle H. Lamberto C. Mollard P. Richalet J. P. (2010). Exercise with hypoventilation induces lower muscle oxygenation and higher blood lactate concentration: role of hypoxia and hypercapnia. Eur. J. Appl. Physiol. 110, 367–377. 10.1007/s00421-010-1512-9 20503056

[B38] Woorons X. Gamelin F. X. Lamberto C. Pichon A. Richalet J. P. (2014). Swimmers can train in hypoxia at sea level through voluntary hypoventilation. Respir. Physiol. Neurobiol. 190, 33–39. 10.1016/j.resp.2013.08.022 24012989

[B39] Woorons X. (2014). Hypoventilation training, push your limits! 1st ed. ARPEH, Lille.

[B40] Woorons X. Mucci P. Richalet J. P. Pichon A. (2016). Hypoventilation training at supramaximal intensity improves swimming performance. Med. Sci. Sports Exerc. 48, 1119–1128. 10.1249/MSS.0000000000000863 26741118

[B41] Woorons X. Mucci P. Aucouturier J. Anthierens A. Millet G. P. (2017). Acute effects of repeated cycling sprints in hypoxia induced by voluntary hypoventilation. Eur. J. Appl. Physiol. 117, 2433–2443. 10.1007/s00421-017-3729-3 29032393

[B42] Woorons X. Millet G. P. Mucci P. (2019a). Physiological adaptations to repeated sprint training in hypoxia induced by voluntary hypoventilation at low lung volume. Eur. J. Appl. Physiol. 119, 1959–1970. 10.1007/s00421-019-04184-9 31286240

[B43] Woorons X. Dupuy O. Mucci P. Millet G. P. Pichon A. (2019b). Cerebral and muscle oxygenation during repeated shuttle run sprints with hypoventilation. Int. J. Sports Med. 40, 376–384. 10.1055/a-0836-9011 30900226

[B44] Woorons X. Billaut F. Vandewalle H. (2020). Transferable benefits of cycle hypoventilation training for run-based performance in team-sport athletes. Int. J. Sports Physiol. Perf. 27, 1103–1108. 10.1123/ijspp.2019-0583 32106076

[B45] Woorons X. Lemaitre F. Claessen G. Woorons C. Vandewalle H. (2021a). Exercise with end-expiratory breath holding induces large increase in stroke volume. Int. J. Sports Med. 42, 56–65. 10.1055/a-1179-6093 32842157

[B46] Woorons X. Billaut F. Lamberto C. (2021b). Running exercise with end-expiratory breath holding up to the breaking point induces large and early fall in muscle oxygenation. Eur. J. Appl. Physiol. 121, 3515–3525. 10.1007/s00421-021-04813-2 34532775

[B47] Woorons X. Daussin F. Combes A. Mucci P. (2023). Physiological responses to supramaximal running exercise with end-expiratory breath holding up to the breaking point. J. Hum. Kinet. 90, 111–123. 10.5114/jhk/174465 38380296 PMC10875693

